# Thermal deformation compensation scheme to the sub-nanometre level of a piezoelectric offset mirror for MHz repetition rate free-electron laser

**DOI:** 10.1107/S1600577524011469

**Published:** 2025-01-01

**Authors:** Baoning Sun, Qinming Li, Chuan Yang, Kai Hu, Zhongmin Xu, Xiaohao Dong, Weiqing Zhang, Xueming Yang

**Affiliations:** a Dalian Coherent Light Source and State Key Laboratory of Molecular Reaction Dynamics Dalian Institute of Chemical Physics, Chinese Academy of Sciences Dalian People’s Republic of China; b University of Chinese Academy of Sciences Beijing People’s Republic of China; c Institude of Advanced Science Facilities, Shenzhen, People’s Republic of China; d Shanghai Advanced Research Institute, Chinese Academy of Sciences Shanghai People’s Republic of China; e Center for Advanced Light Source, College of Science, Southern University of Science and Technology, Shenzhen, People’s Republic of China; Brazilian Synchrotron Light Laboratory, Brazil

**Keywords:** free-electron laser, thermal deformation compensation, piezoelectric deformable mirror, finite-element modelling

## Abstract

Free-electron laser (FEL) facilities operating at MHz repetition rates can emit lasers with average powers reaching hundreds of watts. Partial absorption of this power induces thermal deformation of a few micrometres on the mirror surface. Such deformation degrades the characteristics of the reflected photon beam, leading to focal spot aberrations and wavefront distortions that fail to meet experimental requirements. A robust method is necessary to correct the mirror surface shape to meet the Maréchal criterion. This paper proposes a thermal deformation compensation scheme for offset mirrors operating at MHz repetition rates using a piezoelectric deformable mirror. The mirror is side-mounted with slots filled with an indium–gallium alloy, which house copper tubes for water cooling. Eighteen groups of piezo actuators are symmetrically attached to the top and bottom surfaces. The scheme incorporates finite-element analysis for simulation and post-processing verification, utilizing a differential evolution (DE) algorithm for global optimization. The DE algorithm effectively addresses the voltage constraints that the traditional singular value decomposition algorithm cannot handle. Under an X-ray wavelength of 1 nm, the peak-to-valley (PV) height error of the mirror was reduced from 1340.8 nm to 1.1 nm, and the root-mean-square (RMS) height error decreased from 859.1 nm to 0.18 nm. The slope error was corrected to 154 nrad PV and 24 nrad RMS. Significant results were also achieved at an X-ray wavelength of 3 nm. Wave-optics simulations verified the reliability of this approach, and effects on key mirror parameters and conditions were systematically analysed.

## Abstract 2

A compensation scheme has been developed to control thermal deformation of offset mirrors used in MHz repetition rate free-electron lasers to sub-nanometre level. This approach employs a piezoelectric deformable mirror, utilizes finite-element analysis for simulation and post-processing verification, and integrates a differential evolution algorithm for global optimization.

## 1. Introduction

High-repetition-rate free-electron lasers (FELs), such as Linac Coherent Light Source II (LCLS-II) (Galayda, 2014 ▸), European X-ray Free Electron Laser (European XFEL) (Altarelli, 2015 ▸), the Free Electron Laser in Hamburg (FLASH) (Rossbach *et al.*, 2019 ▸) and Shanghai High Repetition Rate XFEL and Extreme Light Facility (SHINE) (Zhao *et al.*, 2018 ▸), possess unique characteristics like high average power and high coherence. These facilities serve as unprecedented research tools for advanced scientific investigations, including ultrafast reaction dynamics (Fang *et al.*, 2014 ▸), material structure analysis (Takaba *et al.*, 2023 ▸) and high-resolution imaging and spectroscopy (Choi *et al.*, 2024 ▸). They also aim to provide sub-micrometre and nanometre-sized focal spots with high-quality wavefronts, imposing stringent requirements on reflection mirrors to efficiently transport photon beams (Mimura *et al.*, 2010 ▸). Mirrors in practical use can exhibit various imperfections, such as polishing defects, gravity-induced sag, clamping strain, misalignment, vacuum force distortions and heat-load-induced thermal deformations (Alcock *et al.*, 2013 ▸; Alcock *et al.*, 2015 ▸). These imperfections can cause aberrations including beam broadening, diffraction, defocusing and wavefront distortions, thus challenging the beamline’s performance.

High-repetition-rate FELs experience significant heat-load-induced thermal deformations, which are primary factors affecting the surface shape of offset mirrors (Xu *et al.*, 2024 ▸). To address these issues and achieve flexible control of the surface shape, considerable efforts have been made in the field of active and adaptive optics. Unlike passive optics with fixed surface shapes and contact-cooled structures (Cai *et al.*, 1998 ▸; Wang *et al.*, 2022 ▸), active and adaptive optics offer more degrees of freedom and can be adjusted to accommodate various beamline configurations. Beamlines typically use three categories of such optics: electric heating mirrors, mechanically bendable mirrors and piezoelectric deformable mirrors. The resistive element adjustable length (REAL) model exemplifies electric heating mirrors. This model combines cooling with spatially variable auxiliary electric heating to achieve precise figure control, demonstrated by its effectiveness in LCLS-II (Zhang *et al.*, 2015 ▸; Cocco *et al.*, 2020 ▸). A hybrid shape control method using mechanically bendable mirrors has also been developed, offering another approach (Zhang *et al.*, 2024 ▸). However, electric heating mirrors generate only positive power, and mechanically bendable mirrors rely on bending motors solely for cylindrical or elliptical figures, limiting their flexibility. Piezoelectric deformable mirrors offer a versatile solution by applying voltage in two directions, enhancing their correction capability. This enables adjustable surface shapes, including Gaussian, sinusoidal and arbitrary figures, providing an effective solution for compensating thermal deformations (Yang *et al.*, 2012 ▸).

The piezoelectric deformable mirror integrates a reflective mirror with piezoelectric ceramics, typically using lead zirconate titanate (PZT) due to its large piezoelectric constants. It utilizes the inverse piezoelectric effect of the material to induce local contraction or elongation when voltage is applied, thereby achieving flexible control of the mirror surface shape (Alcock *et al.*, 2013 ▸; Cocco *et al.*, 2022 ▸). Research on piezoelectric deformable mirrors began in the 1990s. Early prototypes commonly featured a sandwich structure such as Si–PZT–PZT–Si with a metallic coating deposited on the upper surface. Due to processing constraints, the maximum length of such sections was limited to 150 mm, necessitating the assembly of multiple sections to form longer mirrors. European Synchrotron Radiation Facility (ESRF) developed representative 150 mm monolithic mirrors and 750 mm segmented mirrors for one-dimensional nano-focusing (Signorato *et al.*, 1998 ▸). This type of deformable mirror exhibits significant junction effects, making it less commonly used (Alcock *et al.*, 2013 ▸). Over the past decade, advancements have been made in forming piezo actuators by depositing conductive metal electrodes on PZTs, which are then bonded to a silicon substrate. These actuators, typically ranging between 8 and 32 per mirror, are controlled by applying voltage to adjust the mirror’s shape. SPring-8 developed an 80 mm mirror with piezoelectric ceramics glued to the top and bottom of the optical surface to achieve diffraction-limited focusing (Mimura *et al.*, 2010 ▸). Diamond and Thales-SESO developed a 640 mm mirror with piezoelectric ceramics bonded to the side faces to optimize the surface shape and improve wavefront quality (Alcock *et al.*, 2015 ▸; Sutter *et al.*, 2022 ▸). European XFEL adopted this design and created a 950 mm mirror with an indium–gallium (In–Ga) cooling structure to achieve thermal deformation compensation (Yang *et al.*, 2012 ▸; Vannoni *et al.*, 2016 ▸).

**Figure 1:**
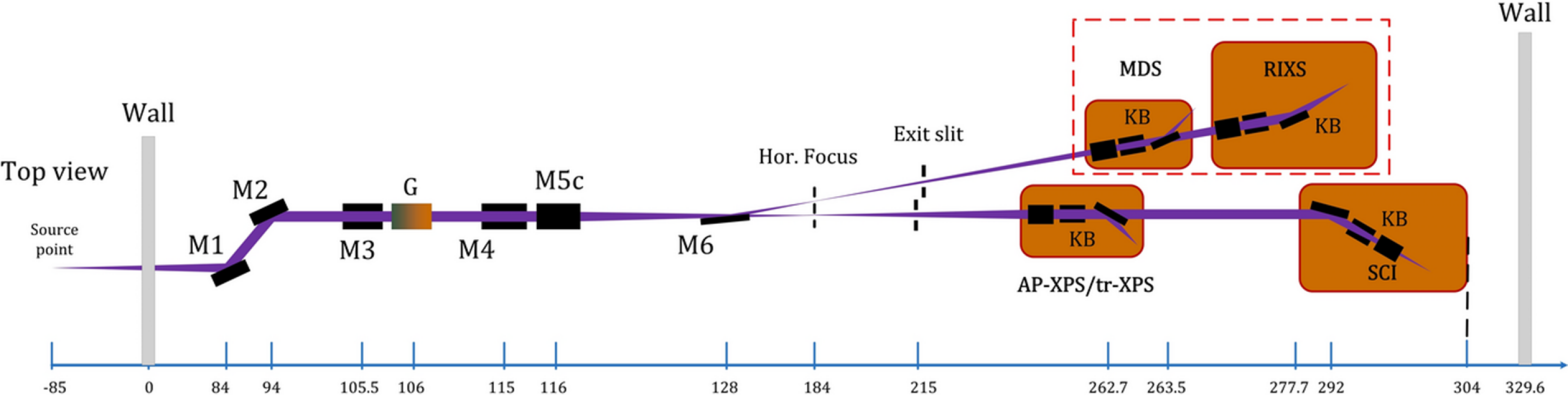
Optical layout of the FEL-1 beamline at S^3^FEL. M1 and M2 form the primary offset mirror group. M3 and G, M4 and M5c are configured for monochromatic and ultrafast endstations, with switchable functionality. M6 serves as a deflecting mirror for FEL beam switching. KB mirror systems at each end station allow for micro-focusing.

Shenzhen Superconducting Soft X-ray Free Electron Laser (S^3^FEL) is a high-repetition-rate X-ray FEL facility currently under construction in China (Wang *et al.*, 2023 ▸). This facility consists of a superconducting linear accelerator with an electron beam energy of 2.5 GeV and a repetition rate of 1 MHz, covering a photon wavelength range of 1 nm to 30 nm. The first phase of the project includes three beamlines, with FEL-1 operating in self-amplified spontaneous emission (SASE) mode from 1 nm to 3 nm. FEL-1 includes four endstations: the Multi-Dimensional Scattering station (MDS), the Resonant Inelastic X-ray Scattering station (RIXS), the Surface Ambient-Pressure and Time-Resolved X-ray Photoelectron station (AP-XPS/tr-XPS) and the Spectroscopy and Coherent Diffraction Imaging station (SCI). Fig. 1 ▸ illustrates the optical layout of the FEL-1 beamline, where M stands for mirror, G for grating and KB for Kirkpatrick–Baez mirror. According to the Maréchal criterion, for a single mirror within this beamline, the root mean square (RMS) height error should be less than 0.9 nm and the RMS slope error should be less than 100 nrad, which approaches the limits of current polishing techniques (Cocco, 2015 ▸).

In this article, we propose a thermal deformation compensation scheme for a reflective mirror operating at MHz repetition rates using a piezoelectric deformable mirror. The offset mirror M1 in the FEL-1 beamline at S^3^FEL is used as an example. The mirror is based on the design by Diamond and Thales-SESO, incorporating the concept used in the European XFEL, where the second mirror compensates for the thermal deformation of the offset mirror. We applied a differential evolution algorithm based on global optimization to obtain the desired constrained control voltages. Finite-element analysis (FEA) was conducted using *ANSYS Workbench* software (Ansys, 2023 ▸) for computation and post-processing verification. This approach demonstrates that piezoelectric deformable mirrors exhibit an excellent capability in correcting thermal deformations in high-repetition-rate FELs. The correction results, in terms of both surface height error and slope error, satisfy operational requirements. Wave-optics simulations further confirm that the beam quality remains unaffected.

## 2. Mirror model

### 2.1. Mirror design

**Figure 2:**
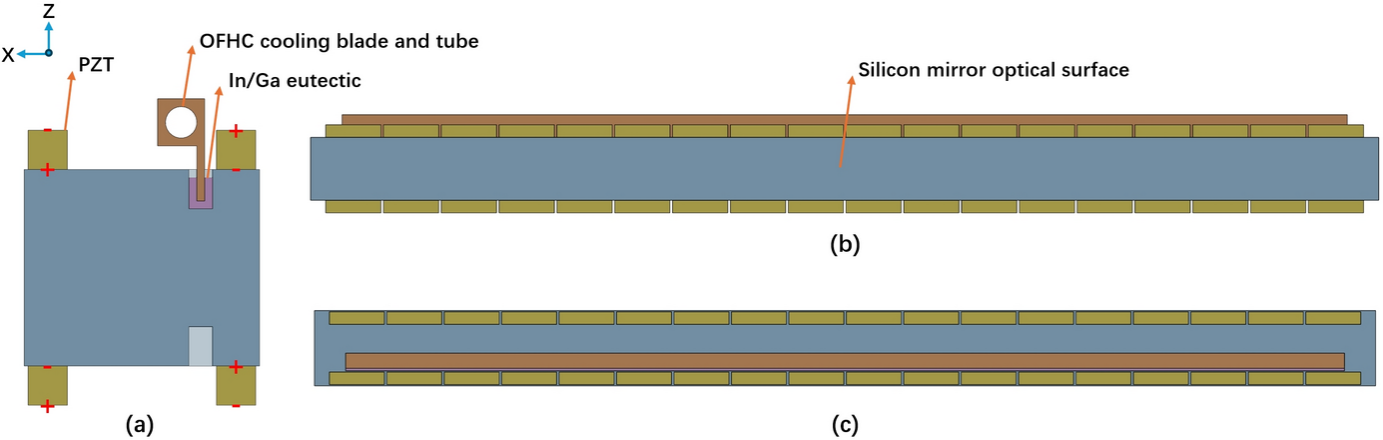
Graphic model of the M1 mirror. (*a*) Cross-section view with voltage directions of piezo actuators and coordinate; (*b*) front view; (*c*) top view. Slots on the mirror’s top surface house the In–Ga eutectic and OFHC cooling tube and blade. Four sets of PZT ceramics are applied with alternating positive and negative voltages.

The offset mirror M1 is the first optical component in the FEL-1 beamline. It is a plane reflective mirror with dimensions of 850 mm × 50 mm × 60 mm, composed of a single crystal silicon substrate coated with B_4_C. Fig. 2 ▸ illustrates a graphic model of M1 with the direction of voltages applied to the piezo actuators. The design features a side-mounted structure, similar to the prototypes at Diamond and European XFEL (Yang *et al.*, 2012 ▸; Alcock *et al.*, 2015 ▸; Vannoni *et al.*, 2016 ▸). The mirror incorporates 18 groups of piezo actuators attached symmetrically to the top and bottom surfaces. Each PZT measures 44 mm × 10 mm × 10 mm, separated by a 2 mm gap. Gold films are deposited on both sides of the PZTs as conductive electrodes to form the piezo actuators. By alternately applying positive and negative voltages to the electrodes, the mirror can be manipulated to produce the desired deformation. Near the reflective side of both the top and bottom surfaces, slots 10 mm deep and 6 mm wide are carved. The top slot is filled with a liquid In–Ga eutectic, and an oxygen-free high-conductivity copper (OFHC) cooling tube and blade are partially immersed to dissipate absorbed power from the FEL beam via water cooling. The surface of the OFHC cooling tube and blade is nickel-plated to inhibit intermetallic reactions with the In–Ga alloy and to improve oxidation resistance. This setup prevents the transmission of cooling tube vibrations caused by water flow to the mirror body, enhancing the stability of the mirror. The slots on both sides ensure the symmetry of the piezoelectric response, while the slot on the bottom also serves as a mounting structure for the mirror.

The finite-element model of M1 was constructed in *ANSYS Workbench*, with specific parameters detailed in Table 1 ▸. PZT exhibits orthotropic elasticity and is polarized along the *Z*-axis. The film coefficient of convection on the inner surface of the OFHC is 5 × 10^−3^ W mm^−2^ °C^−1^. The contact thermal conductance between the mirror, In–Ga eutectic and OFHC is set to 0.15 W mm^−2^ °C^−1^. The ambient temperature is set to 22°C. Since *ANSYS Workbench* does not support direct simulation of piezoelectric materials, the SOLID226 coupled field solid element was selected for PZT through APDL commands to analyse the thermal–piezoelectric–mechanical behaviour, while the element properties of other structures remain preset. Additionally, the glueing layer between the PZT and the Si substrate was ignored because it has a negligible effect on the mirror around the footprint (Yang *et al.*, 2012 ▸).

### 2.2. Heat boundary conditions

**Figure 3:**
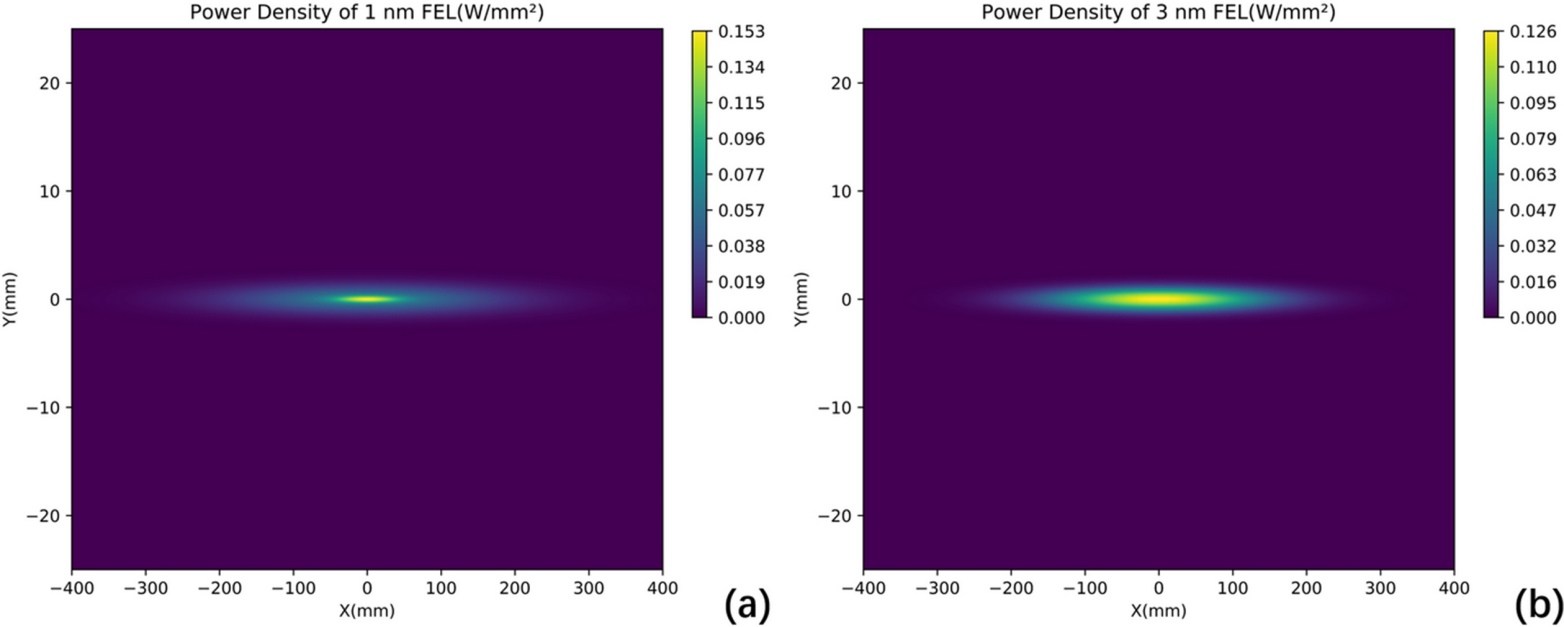
Power density distribution on the M1 mirror at different wavelengths: (*a*) 1 nm; (*b*) 3 nm. The Gaussian distribution displays a smaller footprint with higher peak power density at 1 nm, and a larger footprint with lower peak power density at 3 nm.

**Figure 4:**
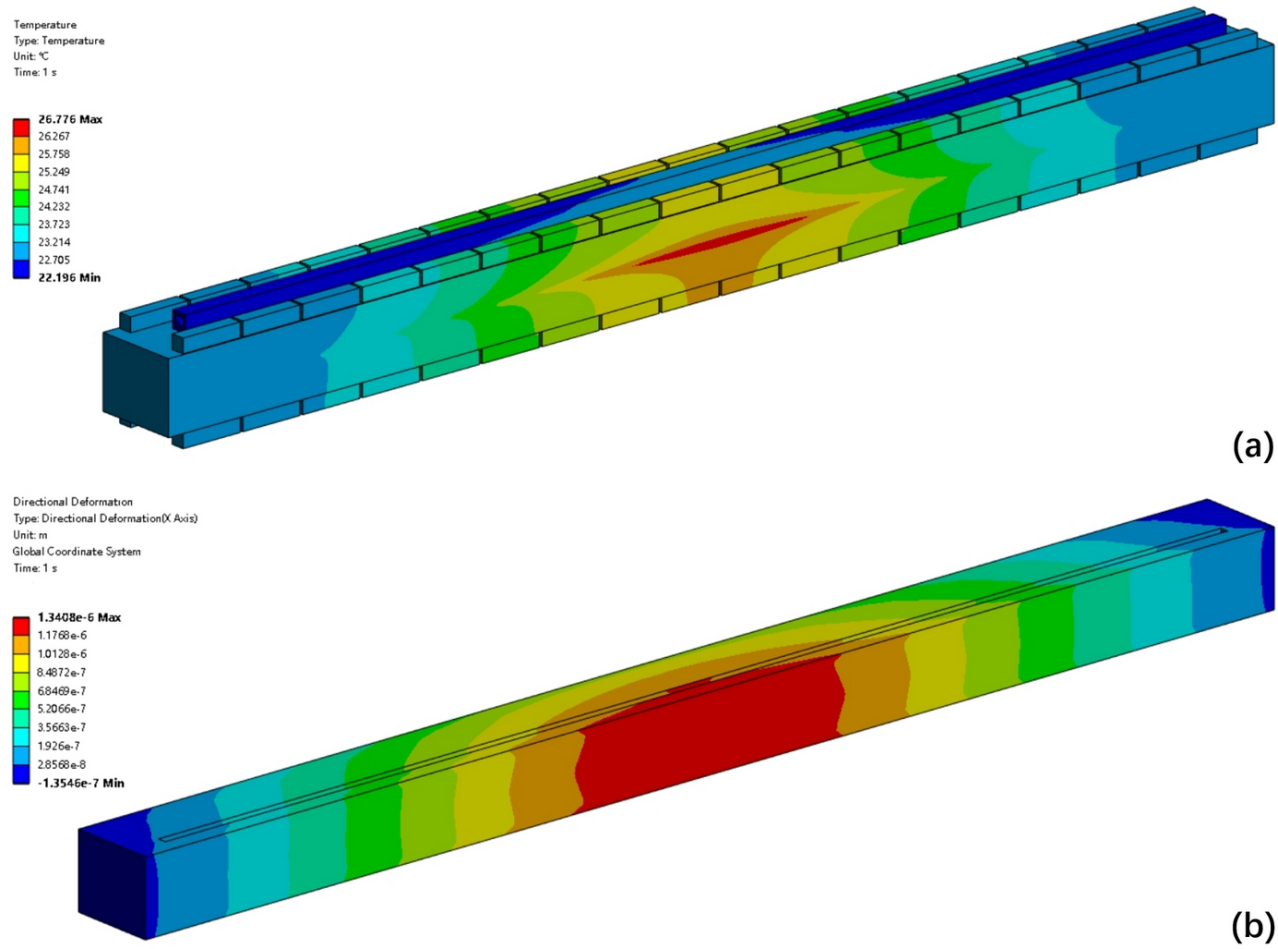
FEA results with absorbed power at 1 nm X-ray wavelength. (*a*) Temperature distribution; (*b*) directional deformation distribution. Temperature distribution corresponds to the power density profile, with lower temperatures near the cooling area and higher on the opposite side. Directional deformation is highest at the mirror centre, gradually decreasing toward the edges.

**Figure 5:**
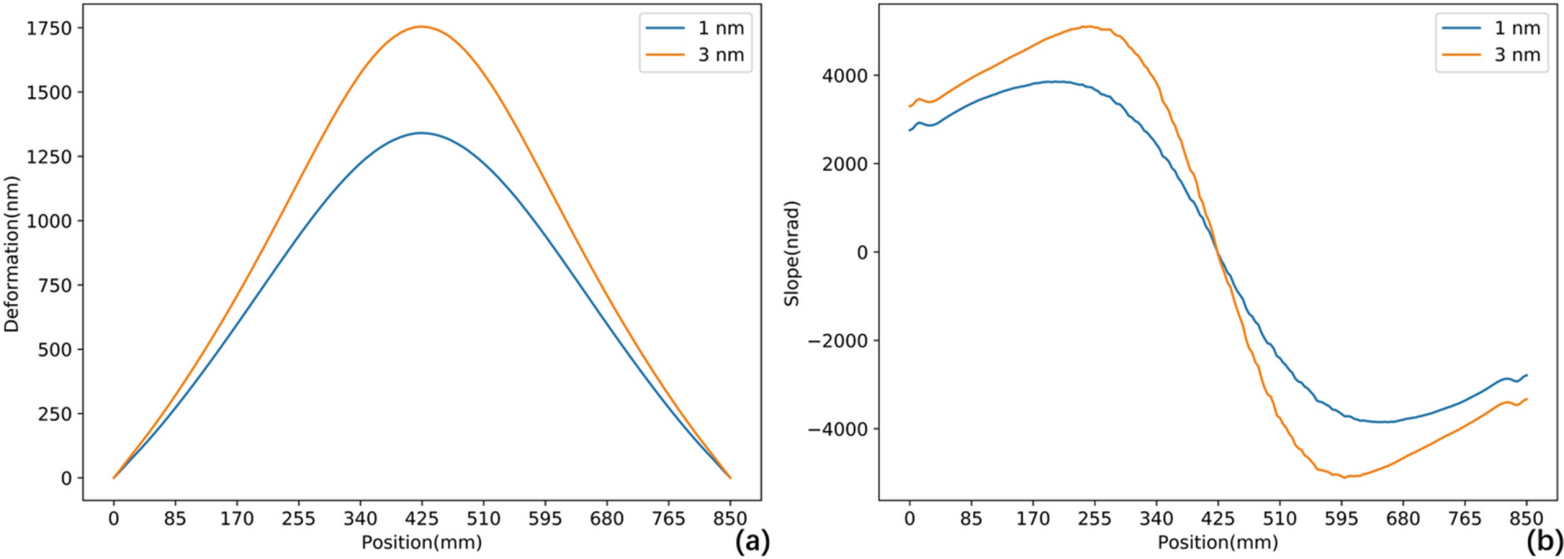
FEA results of the centreline thermal deformation. (*a*) Height; (*b*) slope. Height displays a Gaussian profile, with slope as its first derivative.

As an offset mirror, M1 operates at a grazing incidence angle of 7 mrad. This configuration ensures that the FEL beam has a large footprint on the mirror surface, minimizing single-pulse damage and extreme thermal load. For the X-ray wavelength of 1 nm and 3 nm, the absorption efficiency is 2.6% and 5.3%, respectively (Henke *et al.*, 1993 ▸). The absorbed power is 50 W at 1 nm and 60 W at 3 nm under 1 MHz conditions. Fig. 3 ▸ and Table 2 ▸ provide detailed information on the footprint and absorbed power, as well as their distributions. The power density distribution is incorporated into the FEA model. Taking the 1 nm case as an example, the results are shown in Fig. 4 ▸. Both temperature and directional deformation are primarily concentrated in the centre of the mirror. Due to the elongated shape of the beam footprint, the deformation along the centreline in the meridional direction is most pronounced and has been selected for further analysis, as shown in Fig. 5 ▸. Detailed results, including the 3 nm case, are presented in Table 3 ▸. The irregular deformation, combined with the complex thermal effects induced by the FEL at varying wavelengths and pulse energies, reveals the limitations of conventional mechanical benders and water cooling. This underscores the need for a more robust correction method to maintain FEL beam quality.

### 2.3. Piezoelectric response

**Figure 6:**
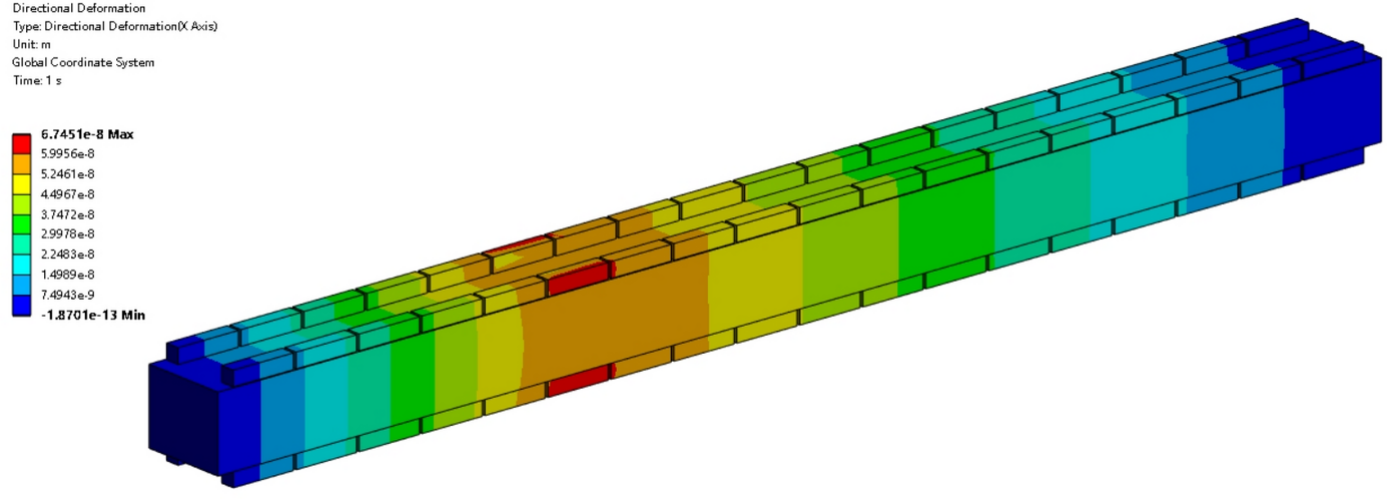
Directional deformation induced by the CH6 channel with 100 V. The piezoelectric deformation in the CH6 channel generates localized deformation in the corresponding mirror region.

**Figure 7:**
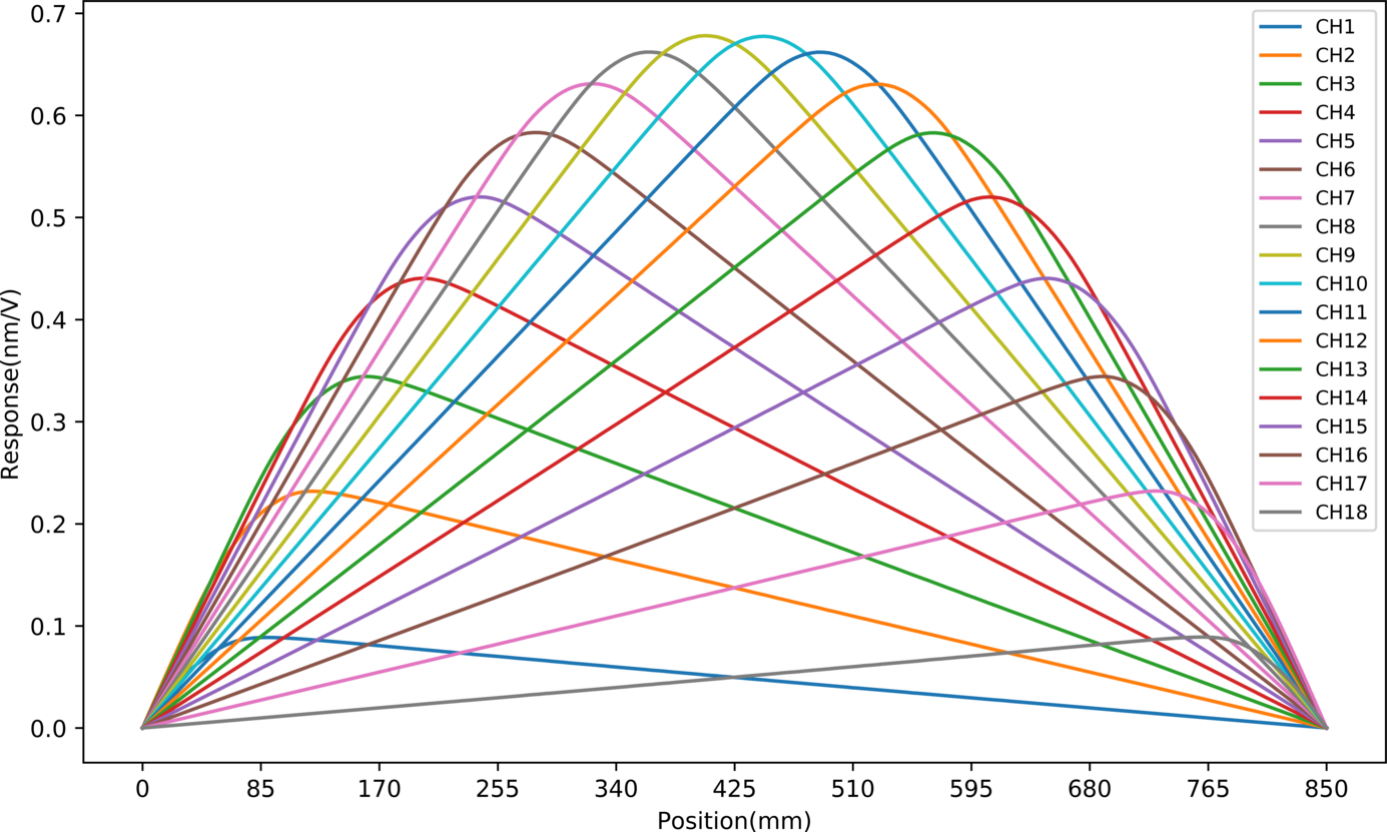
Piezoelectric response function of the M1 mirror. Each electrode generates nanometre-scale deformation per volt, with central electrodes exhibiting a greater response than those at the edges.

Piezoelectric ceramics generate curvature when voltage is applied, resulting in a localized bending effect on the mirror surface. There is a linear relationship between the voltage on each actuator and the induced centreline deformation along the mirror. This relationship can be expressed through the piezoelectric response function (Alcock *et al.*, 2013 ▸). The fundamental compensation principle of piezoelectric deformable mirrors is the linear superposition of different response functions. The M1 mirror is equipped with 18 groups of piezo actuators, each forming a group of electrodes named from CH1 to CH18. Fig. 6 ▸ illustrates the deformation when 100 V is applied to the CH6 channel, with similar deformation principles applicable to the other channels. Fig. 7 ▸ presents the piezoelectric response function of the M1 mirror. The electrodes at the centre produce a stronger response compared with those at the sides, with the maximum response being 0.68 nm V^−1^. By adjusting the voltage of each electrode, flexible and dynamic adjustment of the surface shape can be achieved.

## 3. Figure control method and algorithm

### 3.1. Overview

Controlling mirror surfaces using finite-element models has been a widely studied topic in simulations. Traditional optimization workflows often rely on the built-in algorithms of *ANSYS* (Xu *et al.*, 2023 ▸). Although these algorithms are powerful, they have certain limitations. Firstly, the computational cost is high, especially for complex, multi-objective and multi-variable optimization tasks, as the model must be recalculated each time, leading to slower speeds. Additionally, some algorithms tend to get stuck in local optima, making global optimization challenging. Lastly, parameter adjustment is complex and heavily dependent on the model, with initial conditions and modelling methods significantly influencing the results.

**Figure 8:**
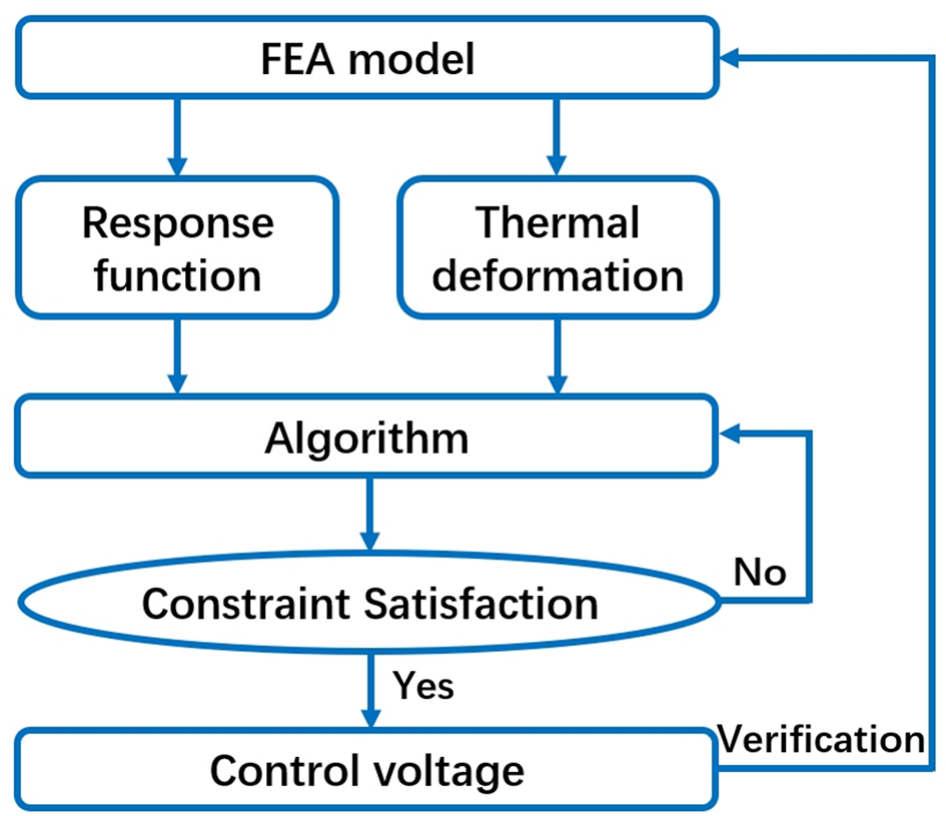
Flowchart of the FEA-based optimization method. This method uses an FEA model to calculate piezoelectric and thermal deformations, along with an algorithm to determine control voltages within specified constraints, and includes self-validation capability.

We developed an FEA-based optimization method to overcome these limitations, as shown in the flowchart in Fig. 8 ▸. Only one finite-element model needs to be constructed. First, the piezoelectric response function and initial thermal deformation are calculated. Next, the control voltage is obtained using an external algorithm, and, finally, the control voltage is applied back to the finite-element model for verification. This optimization process takes place outside of *ANSYS Workbench*, ensuring flexibility and speed.

Following this, precise algorithms can be employed to calculate the required voltage to achieve the desired surface shape. The process used to be challenging because of the high number of electrodes and various constraints. Traditional algorithms mainly include singular value decomposition (SVD) and its derived variants, such as Tikhonov regularization (Huang, 2011 ▸; Vannoni *et al.*, 2014 ▸; Vannoni *et al.*, 2015 ▸). However, these methods have drawbacks, including unacceptable results and slow speeds. In recent years, global optimization algorithms based on iterative computations have shown excellent performance and gained widespread attention and application. These algorithms can iteratively calculate the optimal voltage and easily satisfy various constraints, including voltage limitations (Sumit *et al.*, 2020 ▸). In the following study, the SVD algorithm will be compared with differential evolution (DE) optimization algorithms under identical working conditions. We will use practical examples to underscore the superior capabilities of the DE algorithm.

### 3.2. SVD algorithm

SVD is a widely used method for controlling the surface shape of piezoelectric deformable mirrors. For a mirror with *n* pairs of electrodes and *m* sampling points along the meridional centreline, the relationship between surface shape, response function and voltage can be described by the following equations (Yuan *et al.*, 2021 ▸; Li *et al.*, 2022 ▸),
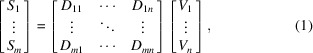
where *S* represents the target surface shape, *D* represents the response function and *V* represents the control voltage. This shows that the surface shape at each point is essentially a linear combination of the responses produced by each electrode under different voltages. The most straightforward way to obtain the solution is through SVD transformation,
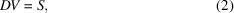

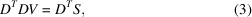

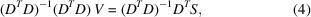

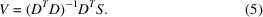
SVD is a purely matrix-based computation method, offering simplicity and speed. However, it has a significant drawback as it can only provide a single solution. To prevent issues such as material fatigue, electric field coupling, and breakdown in PZT piezoelectric ceramics, each individual electrode must have upper and lower voltage limits. Additionally, a limit on the voltage difference between adjacent electrodes should also be set to ensure safe and appropriate operation (Alcock *et al.*, 2015 ▸; Sumit *et al.*, 2020 ▸). For the M1 mirror, the voltage upper and lower limits are set at ±1000 V, and the voltage difference between adjacent electrodes is restricted to no more than ±300 V.

**Figure 9:**
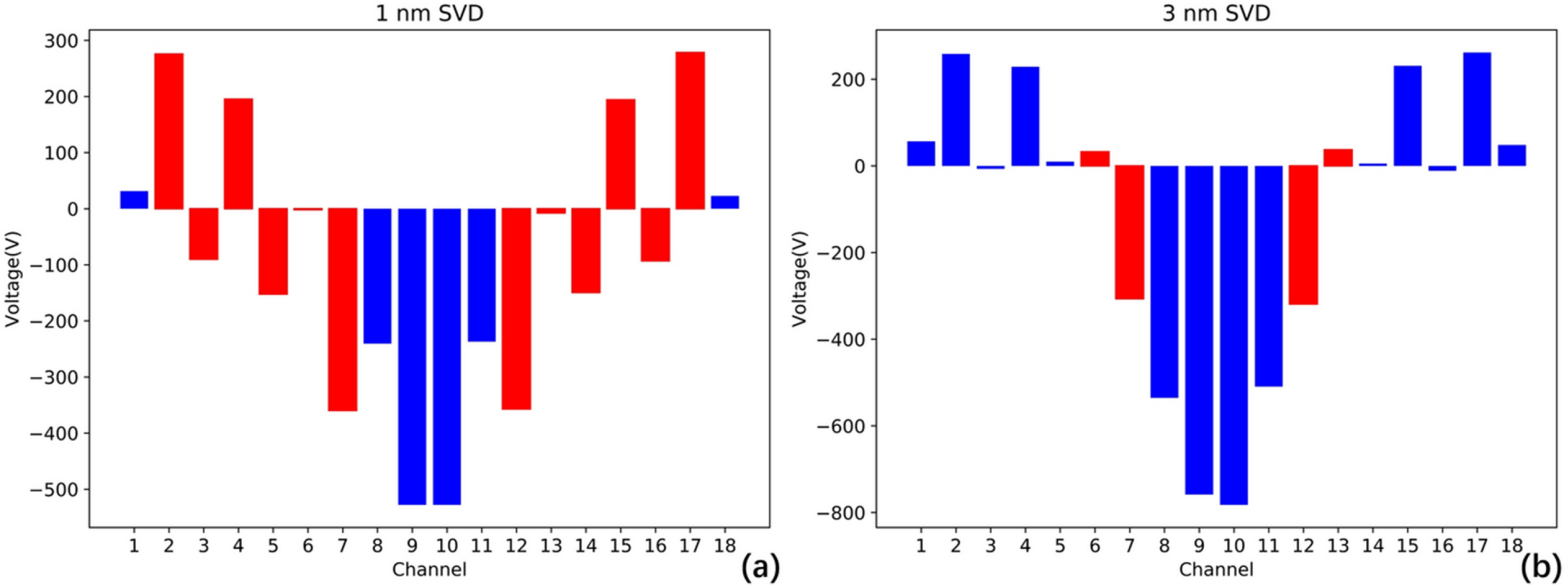
Correction voltages with SVD algorithm at X-ray wavelengths of (*a*) 1 nm; (*b*) 3 nm. Red bars indicate voltages disallowed for exceeding the ±300 V limit between adjacent electrodes, while blue bars represent allowed voltages.

Let us examine the performance of the SVD algorithm using the previously mentioned piezoelectric response function and thermal deformation. Fig. 9 ▸ shows the obtained control voltages, where the blue bars indicate acceptable voltages and the red bars indicate voltages that violate the constraints. It is evident that the SVD algorithm cannot accommodate complex voltage restrictions, presenting a critical flaw in adjusting the piezoelectric deformable mirror’s surface shape and posing potential risks to equipment safety.

### 3.3. DE algorithm

Unlike the SVD algorithm which performs matrix operations to obtain a particular solution, global optimization algorithms are based on iterative optimization processes and can overcome these limitations. Commonly used optimization algorithms include genetic algorithms (GA), simulated annealing (SA), particle swarm optimization (PSO) and DE. These algorithms iteratively calculate the optimal voltage and easily satisfy various voltage constraints. Among these, we chose DE because of its advantages such as simple implementation, insensitivity to parameters, efficient global search capability, robust adaptability and low memory consumption (Price *et al.*, 2005 ▸).

We developed a novel global optimization control scheme for the piezoelectric deformable mirror. The objective function is defined as the RMS difference between the desired surface and the obtained surface on a point-to-point basis. This objective function was then incorporated into a DE algorithm. The algorithm initiates with a set of random voltages and gradually reduces the objective function value through iterative voltage adjustments. The iterative process continues until the imposed constraints are satisfied, typically reaching a specified number of iterations or achieving the desired objective function value. The DE algorithm utilizes the Geatpy library in Python and can obtain the desired optimal solution within a few seconds. It provides the possibility for implementing adaptive control, meeting the requirement for fast and accurate shape adjustment (Jazzbin, 2020 ▸).

**Figure 10:**
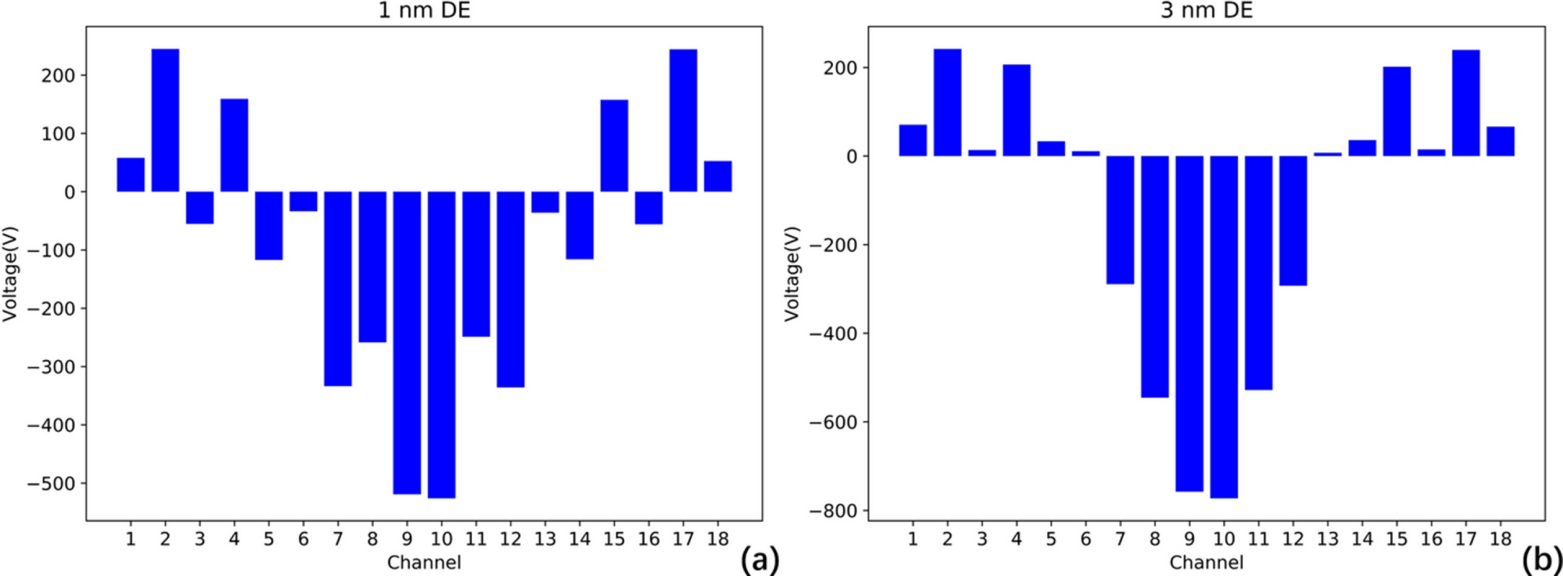
Correction voltages with the DE algorithm at X-ray wavelengths of (*a*) 1 nm; (*b*) 3 nm. All within allowed limits.

The performance of the DE algorithm is shown in Fig. 10 ▸. The overall trend of the voltage is consistent with the results of the SVD algorithm, but all voltages satisfy the constraints mentioned above. The SVD algorithm yields the lowest height error RMS value, which is 0.18 nm, but always not possible for the real system. The DE algorithm, on the other hand, accommodates voltage constraints and achieves an RMS of 0.19 nm, providing nearly identical results. This demonstrates that the DE algorithm is entirely feasible and addresses the limitations of the SVD algorithm. Therefore, all subsequent analysis in this paper will be based on the DE algorithm.

## 4. Results and discussion

### 4.1. Residual surface shape

**Figure 11:**
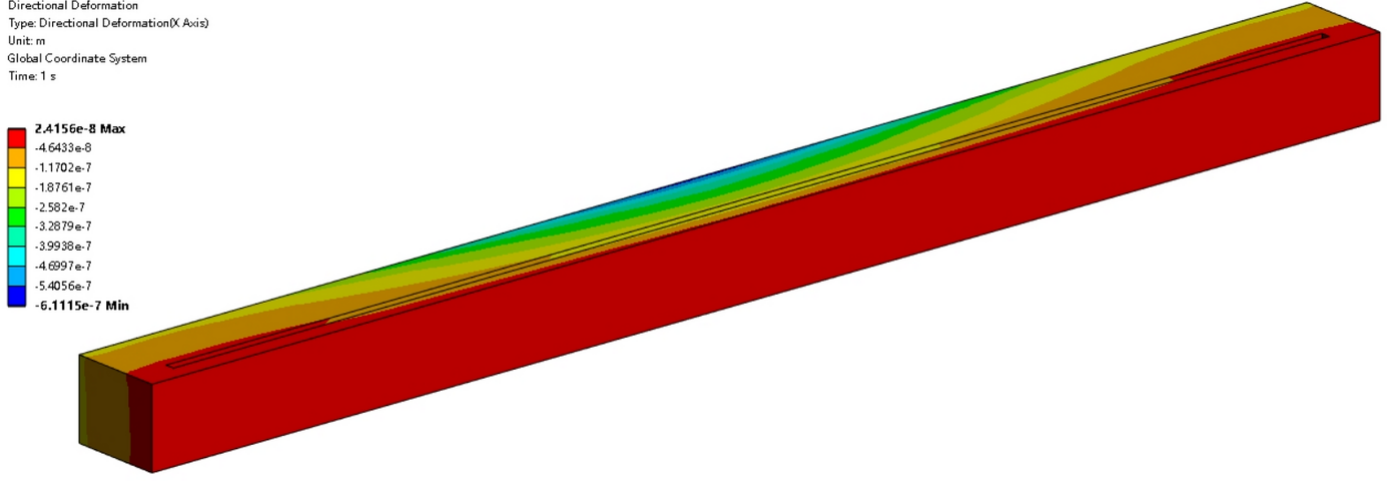
Directional deformation after piezoelectric correction at 1 nm X-ray wavelength. The mirror surface is noticeably flattened after applying control voltages with the DE algorithm.

The control voltages with the DE algorithm are applied to the FEA model for verification. Using the 1 nm case as an example, the FEA results after piezoelectric correction are shown in Fig. 11 ▸. The mirror has been flattened, and the previous thermal deformation has been effectively eliminated.

**Figure 12:**
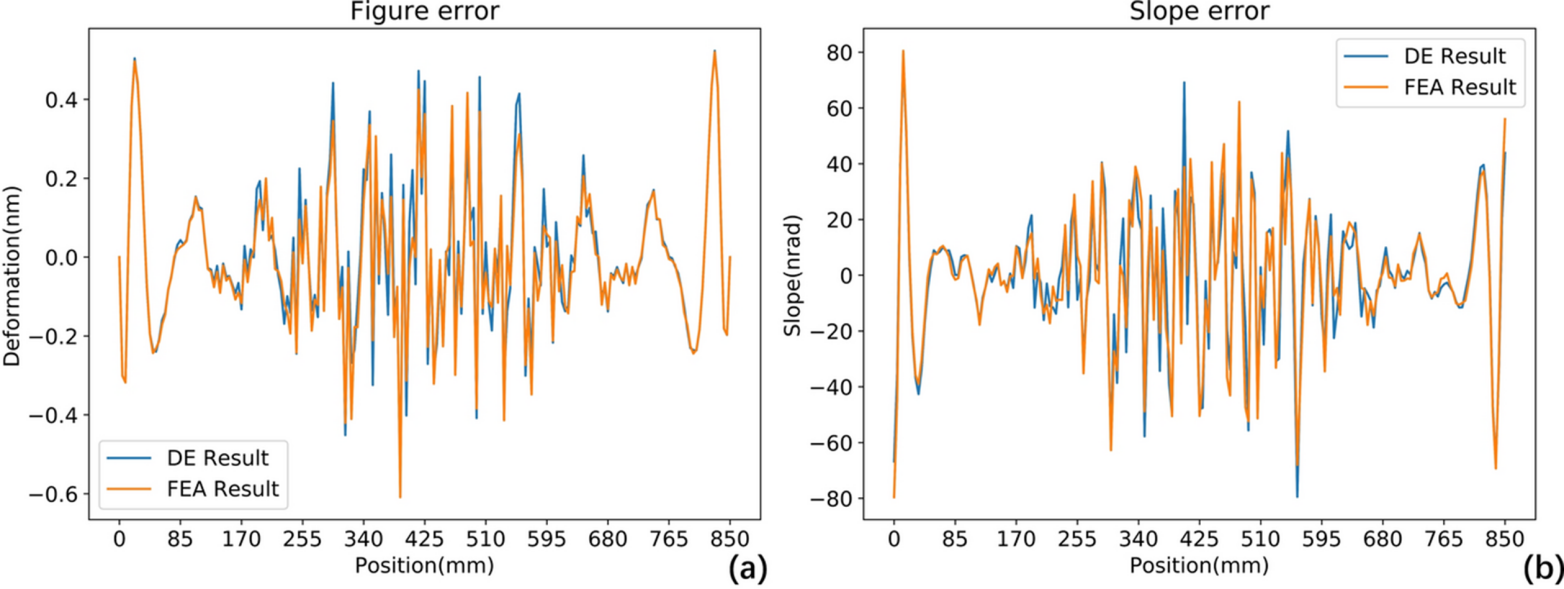
Comparison of centrelines after piezoelectric correction between DE results and FEA results at 1 nm X-ray wavelength. (*a*) Height error; (*b*) slope error. FEA results show good correspondence with DE results, with high-spatial frequency errors attributed to numerical simulation artefacts.

Let us turn our attention back to the centreline of the mirror in the meridional direction. Throughout the correction process we have two sets of correction results. First, the DE algorithm provides an initial result. Second, after FEA verification, we obtain the final result. Fig. 12 ▸ compares these two sets of results in terms of height error and slope error at different wavelengths, showing a high degree of overlap. This demonstrates that the FEA-based optimization method is both feasible and highly reliable.

Table 4 ▸ summarizes the final correction results. For the thermal deformation of 1 nm X-rays, the peak-to-valley (PV) height error was reduced from 1340.8 nm to 1.1 nm, a factor of 1219. The RMS height error was reduced from 859.1 nm to 0.18 nm, a factor of 4773. Meanwhile, the slope error was also significantly corrected, with PV of 154 nrad and RMS of 24 nrad. Comparable results were observed for the 3 nm case. Although it is assumed that the mirror possesses an ideally planar surface, intrinsic surface defects remain even with advanced processing techniques like elastic emission machining (EEM). For example, JTEC, a leading manufacturer, can produce mirrors with a shape error of less than 0.3 nm RMS and a slope error of less than 50 nrad RMS (Jtec, 2024 ▸). The height and slope errors of the offset mirror after piezoelectric correction are lower than these processing errors, confirming the feasibility of this piezoelectric correction method in a MHz beamline.

### 4.2. Wave-optics simulation

**Figure 13:**
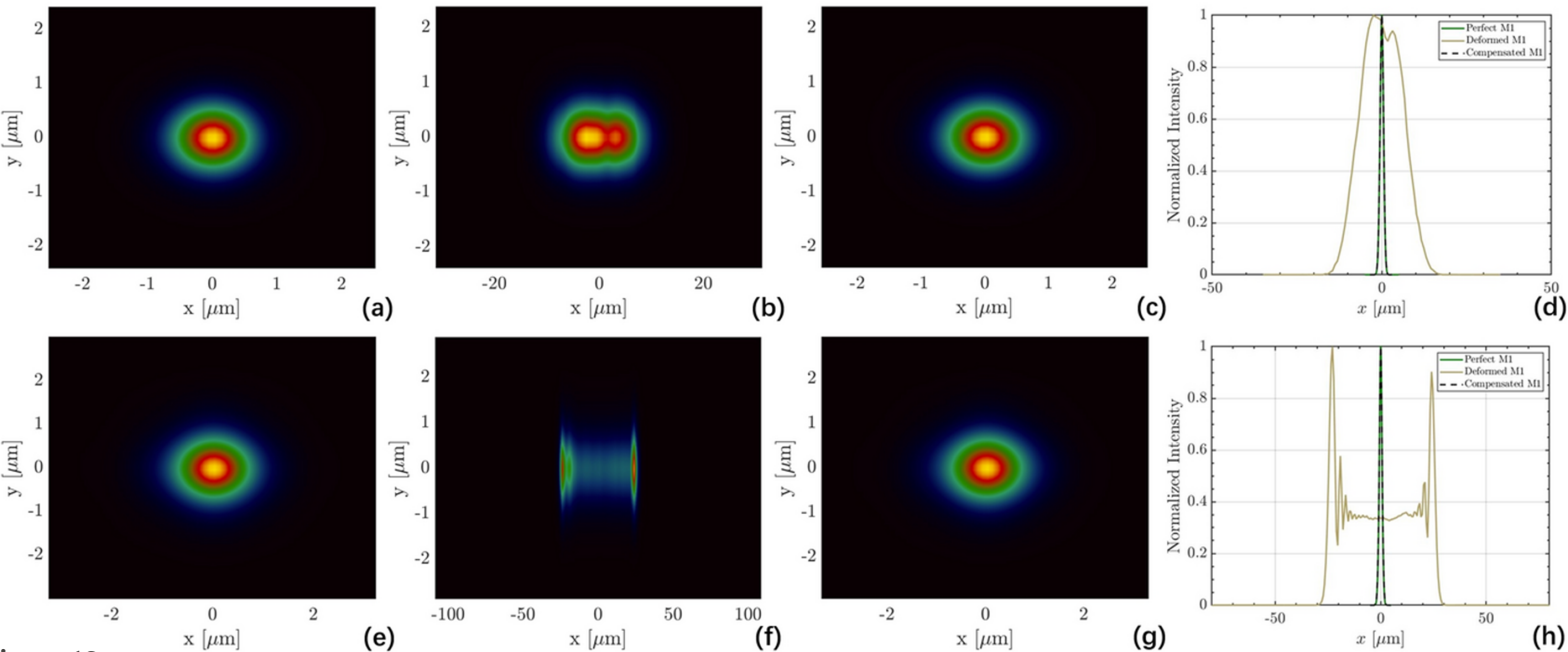
Wave-optics simulation at focal spots for (*a*)–(*d*) 1 nm and (*e*)–(*h*) 3 nm FEL beam passing through (*a*, *e*) perfect M1; (*b*, *f*) M1 with thermal deformation; (*c*, *g*) M1 after shape compensation; (*d*, *h*) intensity distributions. Piezoelectric compensation refines the micrometre-level focus to near-ideal.

Wave-optics simulations were conducted using the *FURION* code to clearly evaluate the impact of the surface shape on the final focal spots before and after compensation (Zhu *et al.*, 2024 ▸). The results are shown in Fig. 13 ▸. When the FEL beam passes through M1 with thermal deformation, while other mirrors remain ideal, significant deformation leads to beam broadening, diffraction and defocusing. However, the focal spots after the compensation are nearly identical to the ideal condition.

### 4.3. Effects on mirror parameters and conditions

**Figure 14:**
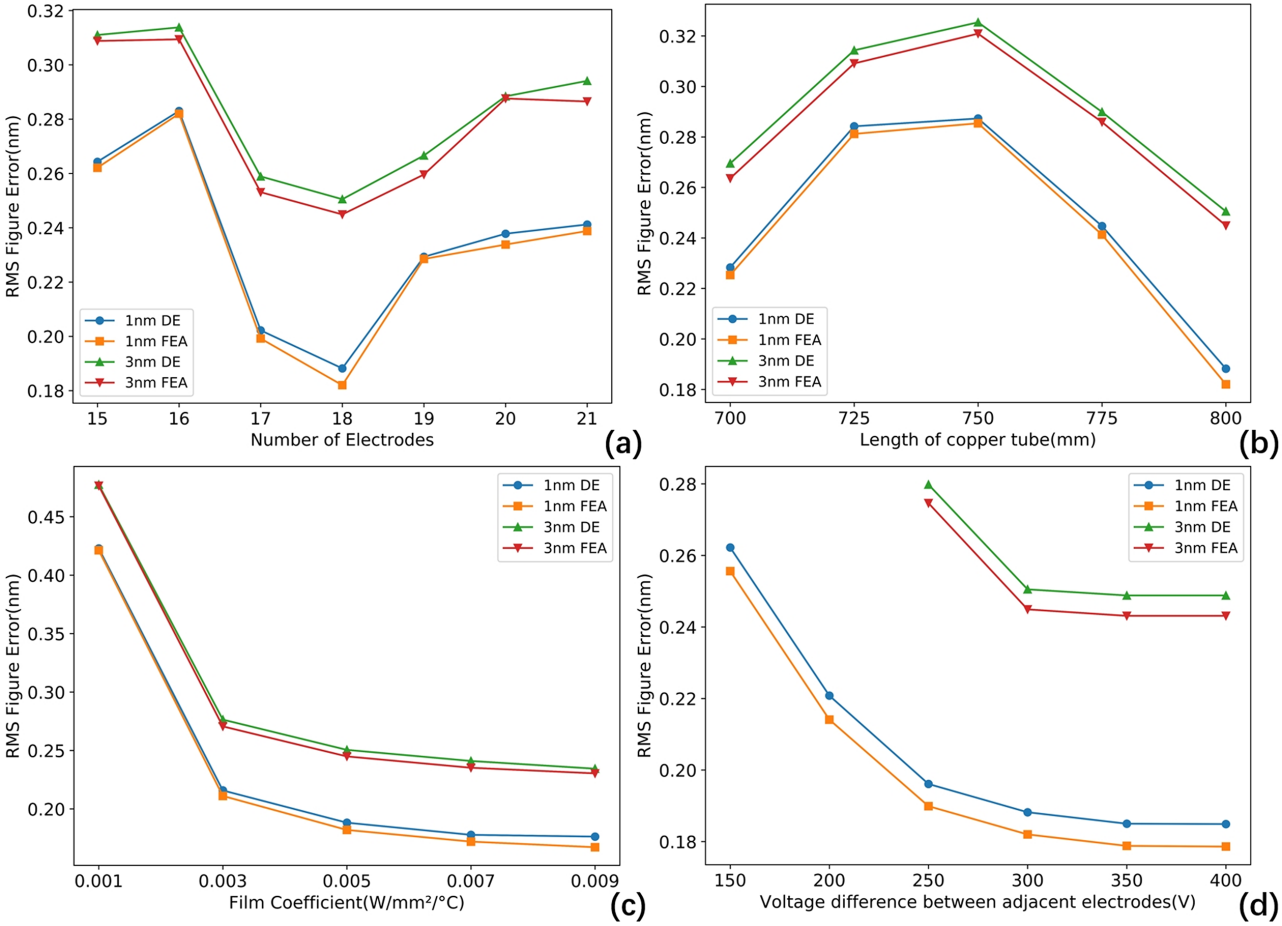
Effect of model parameters on results. (*a*) Number of electrodes; (*b*) length of copper tube; (*c*) film coefficient; (*d*) voltage differences between adjacent electrodes. FEA results align with DE results but are slightly smaller, and 1 nm results are smaller than those at 3 nm.

Finally, we scanned several important parameters in the FEA model, including the number of electrodes, length of the copper tube, film coefficient and voltage differences between adjacent electrodes to conduct a comprehensive evaluation under various engineering conditions, as shown in Fig. 14 ▸.

**Figure 15:**
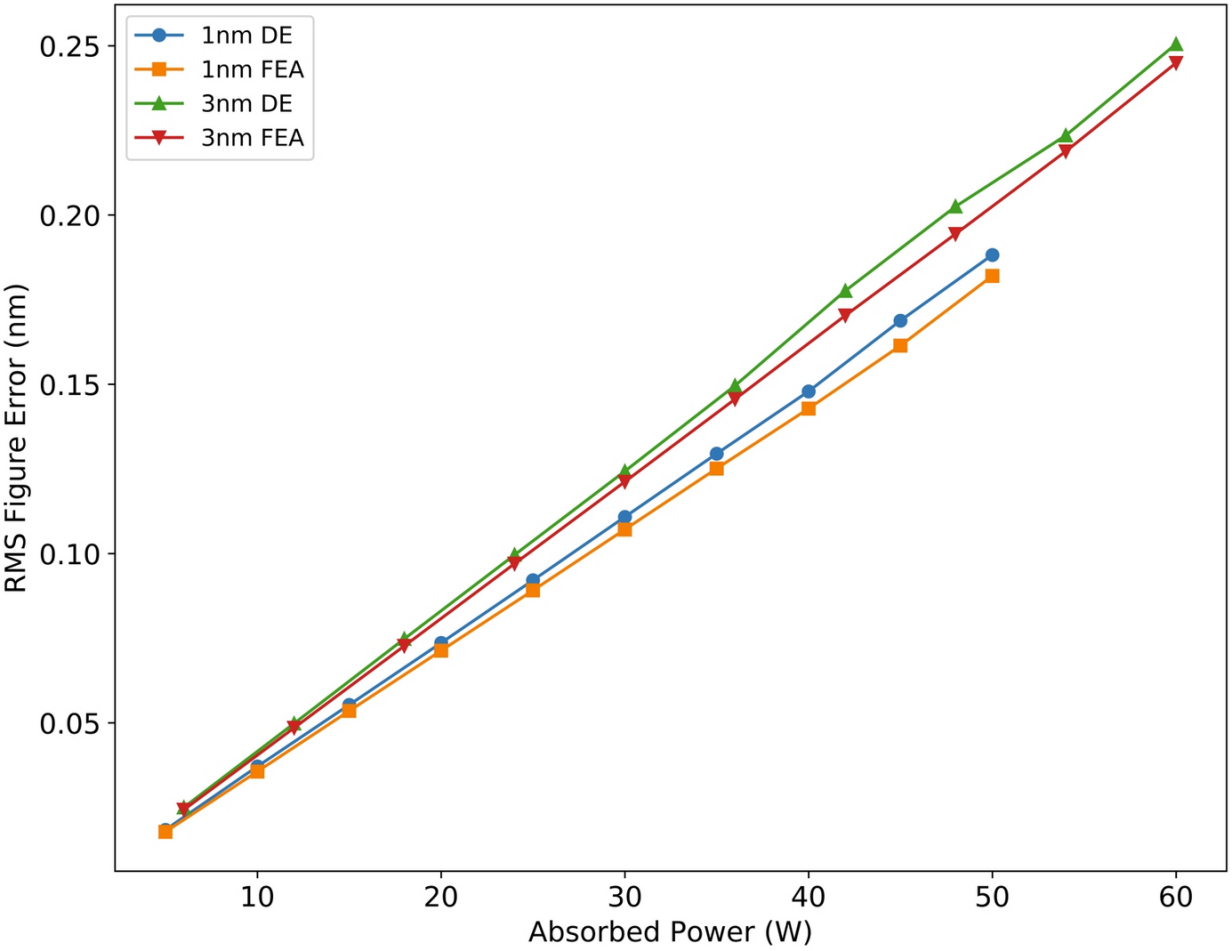
Effect of absorbed power on results. Higher absorbed power leads to a proportional increase in RMS surface error.

Table 5 ▸ presents the geometric parameters of individual PZTs when varying the number of electrodes. The spacing between two PZTs is 2 mm. As the number of electrodes increases, the length of each PZT shortens, reducing the magnitude of the piezoelectric response. Interestingly, more electrodes do not necessarily yield better results, the height error is minimized with 18 groups of electrodes. The length of the cooling copper tube and the heat transfer coefficient primarily affect the initial thermal deformation. Post-correction analysis shows that, when the cooling copper tube is at its full length of 800 mm, the height error is at its lowest. Furthermore, increasing the heat transfer coefficient results in a corresponding reduction in height error. The impact of the upper limit on voltage differences between adjacent electrodes was also evaluated. Less restrictive limits result in lower height error. An upper limit of ±300 V strikes a balance, ensuring safety without significantly increasing the height error. We also evaluated the height error when reducing the repetition rate, thereby decreasing the absorbed power, as shown in Fig. 15 ▸. The height error exhibits a linear relationship, demonstrating that the mirror performs excellently across the entire power range.

The conclusion drawn from the slope error aligns with the above observations and is not shown here. All FEA results are smaller than their corresponding DE results. These comprehensive results can support the engineering selection of piezoelectric deformable mirrors.

### 4.4. Time-based considerations and future outlook

This simulation study ultimately aims to support the development and fabrication of hardware suitable for deployment in high-repetition-rate FEL beamlines. A key challenge is managing dynamic instabilities from both the FEL source and the piezoelectric deformable mirror (Alcock *et al.*, 2019 ▸). Intrinsic fluctuations in FEL systems often arise from variations in electron beam stability, phase shifts within accelerator cavities, disturbances from magnet power supplies, and environmental factors such as temperature changes and vibrations. These fluctuations affect pulse energy and beam power, causing continuous thermal load variations on mirror surfaces and leading to shape changes. Electron-beam-based feedback, incorporating machine-learning algorithms, can optimize FEL lasing to maintain steady photon-induced heat loads on optics, ensuring stable thermal deformation (Sun *et al.*, 2024 ▸). In addition, FEL beamlines are equipped with advanced optical diagnostic systems to monitor and provide feedback on FEL output. For instance, the gas monitor detector enables non-destructive, continuous monitoring to maintain long-term FEL power stability. Instability also arises from inherent characteristics of the piezoelectric deformable mirror, particularly creep in piezoelectric ceramics and hysteresis from resistance in the opto-mechanical holder. Piezoelectric materials exhibit a time-dependent shape drift known as creep, which affects the mirror curvature logarithmically over time. The mechanical holder may create opposing resistive forces that cause gradual, unpredictable shape changes, complicating long-term precision control. Our approach involves using a ZPS interferometer system for continuous nanometre-level surface monitoring to address instabilities from both the FEL source and mirror (Zygo, 2024 ▸). Additionally, a wavefront sensor can provide essential real-time feedback to correct wavefront distortions associated with surface shape adjustments. Artificial intelligence algorithms, trained on historical ZPS and wavefront data, can adaptively correct deviations from creep and hysteresis, enabling predictive adjustments in a closed-loop control system. Minor voltage adjustments address short-term fluctuations over seconds to minutes, while larger adjustments compensate for accumulated effects over hours to days. This approach reflects a developmental trend in piezoelectric deformable mirrors, significantly enhancing stability in high-repetition-rate FEL applications (Alcock *et al.*, 2023 ▸).

## 5. Summary and conclusions

This paper proposes a thermal deformation compensation scheme for offset mirrors in high-repetition-rate FEL beamlines, addressing thermal loads of tens of watts using a piezoelectric deformable mirror. This scheme incorporates finite-element analysis for simulation and post-processing verification and employs a global optimization-based differential evolution algorithm for optimal control. The actual operating conditions of the M1 offset mirror in the FEL-1 beamline at S^3^FEL were used for design verification. The corrected surface error meets stringent requirements under varying wavelengths and is smaller than the current inherent polishing errors of FEL mirrors. Wave-optics simulations further validated the correction performance, and key design parameters were systematically analysed.

This self-consistent methodology provides a framework and valuable reference for developing next-generation high-repetition-rate FEL beamlines. Once the actual piezoelectric deformable mirror is manufactured, offline testing can be conducted in a metrology lab where the experimental surface shape and response function are obtained using a Fizeau interferometer or a long trace profilometer (Zhou *et al.*, 2023 ▸). Control voltages can then be determined without modifying the algorithm. Future online testing and real-time optimization could use a ZPS for continuous nanometre-level monitoring, with a wavefront sensor (*e.g.* Hartmann or fractional Talbot grating) downstream of the mirror. Combined with artificial intelligence algorithms trained on historical data, these tools enable adaptive, predictive control in a closed-loop system. Although simulations show promising results, engineering implementation remains challenging, requiring precise substrate processing, robust mounting structures, effective cooling, and reliable bonding between the PZT layers and substrate. The next step involves engineering a piezoelectric deformable mirror and validating its performance in practical applications.

## Figures and Tables

**Table 1:** Materials parameters for FEA

Material	Density (kg m^−3^)	Youngs modulus (GPa)	Poisson’s ratio	Thermal conductivity (W m^−1^ °C^−1^)	Thermal expansion coefficient (10^–6^ °C^−1^)
Si	2329	112.4	0.28	148	2.5
OFHC	8900	110	0.34	391	17.5
In–Ga	6350	–	–	28	–
PZT	4500	78.6(*X*,*Y*), 62.5(*Z*)	0.29(*XY*), 0.45(*YZ*,*XZ*)	60.5	12

**Table 2:** Working parameters of the M1 mirror

Wavelength (nm)	Footprint (FWHM, mm)	Power (W)	Peak power density (W mm^−2^)
1	135	50	0.153
3	321	60	0.126

**Table 3:** Detailed results of the M1 mirror

Wavelength (nm)	Temperature (°C)	PV height (nm)	RMS height (nm)	PV slope (nrad)	RMS slope (nrad)
1	26.8	1340.8	859.1	8160	3311
3	28.5	1745.5	1087.7	10812	4306

**Table 4:** Summary of FEA correction results

Correct index	Wavelength (nm)	Uncorrected	Corrected	Correction ratio
PV height (nm)	1	1340.8	1.1	1219
	3	1745.5	1.5	1164
RMS height (nm)	1	859.1	0.18	4773
	3	1087.7	0.24	4532
PV slope (nrad)	1	8160	154	53
	3	10812	175	62
RMS slope (nrad)	1	3311	24	138
	3	4306	31	139

**Table 5:** Specifications of each PZT versus the number of electrodes

Number of electrodes	Length of each PZT (mm)	Maximum response (nm V^−1^)
15	54	0.83
16	50	0.77
17	47	0.73
18	44	0.68
19	42	0.65
20	40	0.62
21	38	0.59
